# Histopathologic Review of Previously Negative Prostatic Core Needle Biopsies Following a New Diagnosis of Adenocarcinoma of the Prostate by Core Needle Biopsies: Implications for Quality Assurance Programs

**DOI:** 10.4137/cpath.s581

**Published:** 2008-09-16

**Authors:** Jay Patel, Lester J. Layfield

**Affiliations:** 1 Resident in Department of Pathology, University of Utah School of Medicine and ARUP Laboratories, Salt Lake City, Utah; 2 Professor and Head, Division of Anatomic Pathology, Department of Pathology, University of Utah School of Medicine and ARUP Laboratories, Salt Lake City, Utah

**Keywords:** quality assurance, prostate, core needle biopsy, diagnostic errors

## Abstract

Programs for quality assurance are increasingly important in surgical pathology. Many quality assurance (QA) techniques for surgical pathology were adopted from procedures introduced in cytopathology. Surgical pathology specimens have diminished in size such that the majority of diagnostic biopsies of prostatic lesions are now core needle biopsies. These specimens raise issues similar to those of cytology specimens, including concerns regarding adequacy and the representative nature of the biopsy. Due to sample size, some neoplasms may not be diagnosed on initial biopsy, raising concerns regarding false negative results. Cytopathologists have instituted QA procedures including review of all previously negative slides received within five years prior to the new diagnosis of high grade squamous intraepithelial lesion or gynecologic malignancy. No such requirement exists in surgical pathology for review of core biopsies.

The Department of Pathology at the University of Utah instituted a QA policy requiring review of prior negative prostatic needle biopsies following a new diagnosis of prostatic adenocarcinoma. We reviewed five years of QA records of prostate needle biopsy review. During this time, nine hundred and fifty-eight core biopsy sets were performed. Two hundred and ninety-five of these contained at least one biopsy with a diagnosis of adenocarcinoma. Two hundred and eight patients had a prior set of prostatic needle biopsies with a diagnosis of adenocarcinoma. The remaining 87 had prior biopsies with either a diagnosis of prostatic intraepithelial neoplasia (23), small atypical acinar proliferation (21) or no evidence of malignancy (43). QA review of these 87 cases revealed two biopsies which revealed foci of adenocarcinoma. Both had been initially diagnosed as no evidence of malignancy. The false negative rate for core biopsy was 0.68%. In an additional twenty-one cases, microscopic foci of atypical small acinar proliferations were found in core biopsies antedating the positive core biopsy (7.1%).

## Introduction

Quality assurance programs have become an important component of laboratory management in Anatomic Pathology. Quality assurance and control programs have long been used for assessment of the technical components of Anatomic Pathology practice. These procedures have only more recently become widely utilized for assessment of the interpretive portion of Anatomic Pathology practice. Quality assurance programs have varied widely in their scope, ranging from mandatory 100% review of Surgical Pathology diagnoses,^16^ to more limited programs such as histologic review of materials obtained from patients referred to tertiary centers for a second opinion or definitive therapy.^3^,^5^ These reviews have documented a small but significant false positive and false negative rate for many specimen types. Epstein et al.^3^ demonstrated the value of second opinion review of outside materials before definitive therapy. Others have confirmed the value of second opinion consultation programs.^8^,^9^,^12^–^15^

Many institutions, including the University of Utah, have quality assurance programs which randomly review approximately 10% of all Surgical Pathology cases and require confirmation by a second pathologist of all malignant diagnoses. The 100% review format allows identification of diagnostic trends among pathologists and allows retrospective institution of training programs and procedures when trends in diagnostic errors are uncovered. One hundred percent prospective review of surgical pathology cases is costly and may prolong turnaround time.^10^,^11^ The method of outside slide review improves the overall predictive value of a positive diagnosis, but reviews only a small percentage of cases and will detect predominantly false positive diagnoses.

Cytopathologists have often taken the lead in developing QA/QC programs in Anatomic Pathology. One innovation originating from cytopathology is the retrospective review of prior cytologic specimens when a new positive diagnosis of high grade dysplasia (cervical intraepithelial neoplasia) is made. The College of American Pathologists, in their inspection protocol requires that cytology laboratories review all prior negative cervical cytologies over a five year period after a new diagnosis of high-grade squamous intraepithelial lesion is made, or a malignant gynecologic case is reported. No similar requirement is currently in effect for Surgical Pathology specimens. Because increasing numbers of breast and prostatic lesions are first investigated by core-needle biopsy, a similar program might be of value. Core-needle biopsies raise similar issues of sampling and interpretation of limited material as are experienced with cytologic specimens. We chose to review prostate core biopsies because they are frequently repeated when initial biopsies are negative and the serum prostatic specific antigen remains elevated. To determine the effectiveness of such a program in uncovering prior false negative diagnoses, we reviewed our five year experience of prostatic core-needle biopsies. Herein we report the results of that review.

## Materials and Methods

The quality assurance records recording the results of the prostate core-needle biopsy review program at the University of Utah Department of Pathology between September 2001 and September 2006 were reviewed. During that period, 958 core-needle biopsy sets (six biopsies each) were performed. Two hundred and ninety-five of these biopsy sets had at least one specimen with a diagnosis of adenocarcinoma and had prior prostate core-needle biopsies. The remaining 663 sets of prostate core needle biopsies either had benign diagnoses or had no prior core needle biopsies. In the QA program, every new diagnosis of adenocarcinoma of the prostate identified on core biopsy was automatically flagged and a computer search for all prior needle biopsies of the prostate for that patient was performed. The slides from the previous core biopsies were obtained and reviewed by a senior surgical pathologist. When necessary, immunohistochemistry for CK903 and CK5/6 was performed. The original and reviewed diagnoses for each of the prior biopsies were recorded along with the size and Gleason score of the carcinoma when present. The name of the pathologist reviewing the prior core biopsy was recorded. While a urologic oncology conference is held biweekly, only positive for malignancy prostate cases are presented and routinely reviewed by a second pathologist. Repeat sets of core needle biopsies were performed due to a “watchful waiting” protocol (prior positive biopsies) or because of a persistently elevated serum prostatic specific antigen level.

Immunohistochemistry for CK5/6 and K903 was performed on one case at the time of second review. The antibodies for CK5/6 were obtained from Chemicon (Temecula, CA) and the antibody was used at a dilution of 1:400. The antibody for K903 was supplied by Enzo (Farmingdale, NY) and applied at a dilution of 1:40. In all cases, biopsies were formalin fixed and paraffin embedded. The diagnostic biopsies were cut at 4 microns and stained with hematoxalin and eosin.

## Results

**Figure 2.**
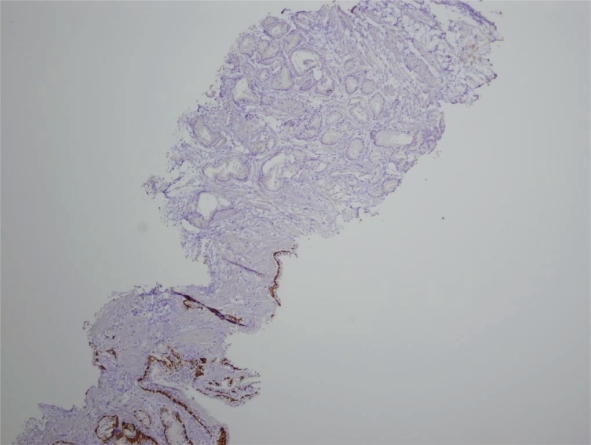
Immunohistochemical staining for CK5/6 demonstrating an absence of basal cells in the focus of adenocarcinoma.

Between September 2001 and September 2006, nine hundred and fifty-eight core-needle biopsy sets (six biopsies each) were examined pathologically. Two hundred and ninety-five of these sets of prostatic biopsies contained at least one core biopsy associated with a diagnosis of adenocarcinoma. Two hundred and eight of these patients had had a prior prostatic core needle biopsy where a diagnosis of adenocarcinoma had been made. The remaining eighty-seven patients had prior sets of biopsies in which at least one biopsy had a diagnosis of prostatic intraepithelial neoplasia (PIN) (23 patients), small acinar proliferation suspicious for carcinoma (21 patients) or no evidence of malignancy (43 patients). Review of the material from these eighty seven patients reveal two biopsies in which needle-core sections revealed diagnostic foci of adenocarcinoma of the prostate. In both cases, the foci of adenocarcinoma were microscopic foci representing less than 5% of core biopsy volume. Both cases were Gleason score 3+3, both in the new index biopsy and the focus discovered on second review (Fig. 1). Immunohistochemistry performed at the time of second review demonstrated an absence of basal cells in the atypical proliferation (Fig. 2). Both cases in which the underdiagnosis had been made, had initially been diagnosed as showing no evidence of malignancy. At the time of the initial false negative core biopsies, the two patients had serum prostatic specific antigen levels of 10.0 ng/ml and 4.4 ng/ml respectively. In this review series, the false negative rate for core-needle biopsy of the prostate was 0.68%. Both false negative diagnoses had been rendered by junior members of the division of Anatomic Pathology. Following the diagnosis of adenocarcinoma, both patients have had at least one additional prostatic needle biopsy showing Gleason score 3+3 adenocarcinoma. Neither patient had been treated by radiation or radical prostectomy. The treating surgeon was notified of the prior false negative diagnosis in both cases. In an additional 21 patients, original biopsies had shown microscopic collections of atypical small acinar proliferations (7.1%). Review of the original biopsy material in these 21 cases showed the atypical foci disappeared on deeper levels precluding the use of immunohistochemistry for the demonstration of the presence or absence of basal cells.

## Discussion

Error reduction has become an increasingly important concern in medical practice. Adequacy of programs for quality control and assurance has become an important issue in diagnostic surgical pathology with additional procedures and programs being encouraged or mandated by societal or government leaders. Numerous studies have shown a significant error rate in diagnostic surgical pathology both between referring and consultant pathologists and among consultant experts.^2^,^4^,^6^,^7^ Reported diagnostic error rates associated with review procedures have varied substantially. Epstein et al.^3^ reported that 1.3% of outside needle biopsies had been incorrectly diagnosed as malignant. Weydert et al.^16^ reported a “major discrepancy rate” of 0.29% between preliminary diagnosis and final staff pathologist diagnosis in a prospective blinded dual review system for general surgical pathology. In a study by Abt et al.,^1^ 5.5% of all diagnoses showed a therapeutically significant discrepancy between a referring institution and the pathology opinion of the treating institution. Similarly high discrepancy rates between consultant and referring pathologists have been reported by others.^2^,^4^,^6^,^7^ From the point of diagnostic accuracy and patient care, a prospective peer review system in surgical pathology as described by Weydert et al.^16^ would appear optimal. However, issues concerning turn-around time and costs mitigate against the universal acceptance of such a policy.^10^,^11^ Interinstitutional reviews as described by Abt^1^ and Epstein^3^ are of value when patients are initially seen at one institution but treated at a second. However, such programs would probably impact a minority of operative patients. Most other QA systems are retrospective and would not be expected to immediately impact the care of the patient whose material was undergoing review. These retrospective systems, however, are of value in that they are useful for documenting diagnostic problems for both individual and groups of pathologists. The systems can lead to modification of practice patterns and focus of continuing education programs. Many institutions perform a retrospective review of ten percent of all Surgical Pathology cases. This system, when random, may be slow to document trends in diagnostic errors in uncommon specimens. When case selection is left to the reviewing pathologists, the tendency to select small, easily reviewable specimens may hinder the effectiveness of the review process in discovering errors in complex or diagnostically difficult lesions. An alternative technique is to focus on specific specimen types where diagnostic errors could have high clinical impact. This focus has been taken by cytopathologists and the College of American Pathologists in their requirement for review of prior negative specimens whenever a new diagnosis of high grade dysplasia or gynecologic malignancy is made on a cervical cytology. This approach has the advantage of focusing on diagnostically difficult areas where false negative diagnoses could have significant clinical impact.

The five year look-back technique used in gynecologic cytology was applied to prostate core-needle biopsies at the University of Utah. Prostate core-needle biopsies were selected for review because of their significant clinical impact when incorrectly read as negative. The small size of the biopsies also makes diagnostic errors more likely than when larger specimens are reviewed. In the past two decades, a majority of prostatic and breast lesions have been investigated by core-needle biopsy. These core-needle biopsies are of relatively small gauge and their interpretation raises issues similar to those seen in cervical cytology. Issues for these small biopsies include the representative nature of the sample and whether or not it contains an adequate amount of pathologic material to allow definitive diagnosis. The QA records of the prostate core-needle biopsy five year retrospective review at the University of Utah demonstrated a 0.68% false negative rate for interpretation of core-needle biopsies. This rate did not include sampling errors, but simply reflected interpretive errors. Both false negative diagnoses were associated with microscopic foci of Gleason score 3 + 3 adenocarcinoma. The foci in both patients represented no more than 5% of the original core-needle biopsy volume. In both cases, the false negative diagnosis had been rendered by a junior member of the division of Anatomic Pathology. While immunohistochemistry for CK5,6 and K903 were used to confirm the presence of adenocarcinoma in the retrospective review process, the senior pathologists performing the review did not require immunohistochemistry for definitive diagnosis.

In an additional twenty-one cases, microscopic foci of atypical small acinar proliferations were found in core-needle biopsies antedating the diagnostically positive core biopsy. Review of these 21 cases confirmed the presence of small atypical acinar structures which did not fulfill stringent diagnostic criteria for adenocarcinoma. In approximately half of these cases, a basal cell layer was demonstrable by immunohistochemistry in the acinar proliferation. This basal cell layer was usually non-continuous, but its presence dissuaded the review pathologist from rendering a definitive diagnosis of adenocarcinoma. In 23 cases, only high grade prostatic intraepithelial neoplasia was detected, and even at subsequent review, knowing that the patient had biopsy proven adenocarcinoma, a definitive diagnosis of adenocarcinoma could not be made by the senior review pathologist.

The five year retrospective review program at the University of Utah demonstrated a false negative interpretive rate of approximately 0.68%. This is higher than the 0.29% clinical major discrepancy rate reported by Weydert et al.^16^ In that study, the precise number of prostate biopsies was not given, but three of the eighteen major discrepancies involved prostate biopsies or TURP specimens. Two of the errors reported by Weydert et al. were underdiagnoses of adenocarcinoma by the initial hot seat fellow. Thus our five year look-back review and Weydert et al.’s prospective review both demonstrating a significant false negative rate for interpretation of prostatic biopsy specimens. Epstein’s report of a 1.3% false positive rate in prostate core-needle biopsy specimens indicates that prostatic needle-core biopsies are difficult to interpret and may represent a specimen type requiring additional review before final sign-out. By itself, the retrospective review process yields valuable information useful for resident teaching, continuing medical education, and the monitoring of practice patterns in pathology groups.

The discovery of a prior false negative diagnosis during QA review raises legal and ethical issues including the need to inform the treating physician and patient. Given the current medicolegal climate, many pathologists may be reticent to undertake QA programs likely to uncover false negative diagnoses, as such cases might result in delay of diagnosis lawsuits. Recent public policy initiatives have been developed with an emphasis on a “guilt free environment” to facilitate the recognition of and reduction in the number of medical errors. QA programs such as the one described here may find increased utility for the recognition of medical errors by the QA process if the medico-legal environment allows a truly “guilt free” approach.

## Figures and Tables

**Figure 1A.**
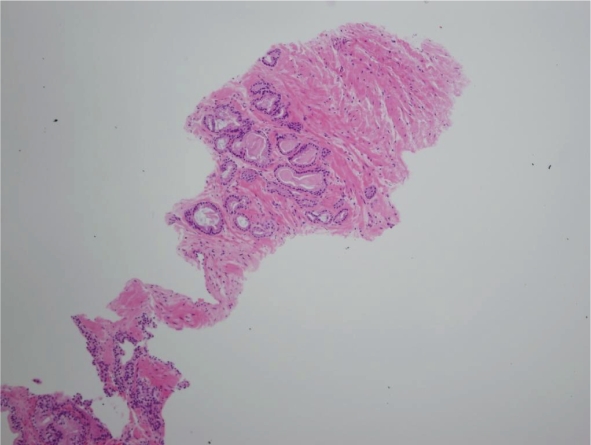
Photomicrograph showing small focus of adenocarcinoma missed at the time of initial diagnosis (H and E).

**Figure 1B.**
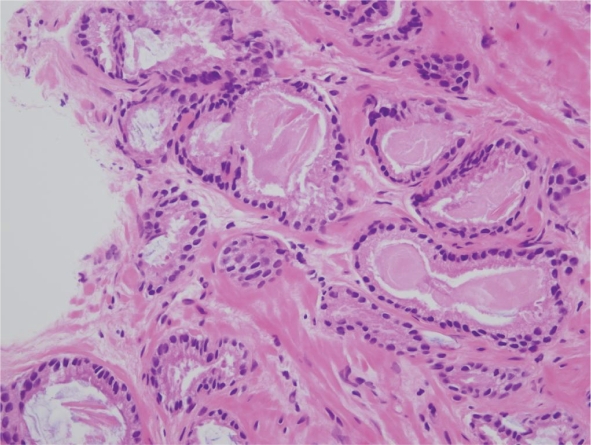
High power view of adenocarcinoma (H and E).
